# Hearing Scenes: A Neuromagnetic Signature of Auditory Source and Reverberant Space Separation

**DOI:** 10.1523/ENEURO.0007-17.2017

**Published:** 2017-03-01

**Authors:** Santani Teng, Verena R. Sommer, Dimitrios Pantazis, Aude Oliva

**Affiliations:** 1 Computer Science and Artificial Intelligence Laboratory, Massachusetts Institute of Technology, Cambridge, MA 02139; 2 Amsterdam Brain and Cognition Centre, University of Amsterdam, 1018 WS Amsterdam, The Netherlands; 3 McGovern Institute for Brain Research, Massachusetts Institute of Technology, Cambridge, MA 02139

**Keywords:** audition, auditory scene analysis, magnetoencephalography, multivariate pattern analysis, reverberation

## Abstract

Perceiving the geometry of surrounding space is a multisensory process, crucial to contextualizing object perception and guiding navigation behavior. Humans can make judgments about surrounding spaces from reverberation cues, caused by sounds reflecting off multiple interior surfaces. However, it remains unclear how the brain represents reverberant spaces separately from sound sources. Here, we report separable neural signatures of auditory space and source perception during magnetoencephalography (MEG) recording as subjects listened to brief sounds convolved with monaural room impulse responses (RIRs). The decoding signature of sound sources began at 57 ms after stimulus onset and peaked at 130 ms, while space decoding started at 138 ms and peaked at 386 ms. Importantly, these neuromagnetic responses were readily dissociable in form and time: while sound source decoding exhibited an early and transient response, the neural signature of space was sustained and independent of the original source that produced it. The reverberant space response was robust to variations in sound source, and vice versa, indicating a generalized response not tied to specific source-space combinations. These results provide the first neuromagnetic evidence for robust, dissociable auditory source and reverberant space representations in the human brain and reveal the temporal dynamics of how auditory scene analysis extracts percepts from complex naturalistic auditory signals.

## Significance Statement

We often unconsciously process echoes to help navigate places or localize objects. However, very little is known about how the human brain performs auditory space analysis and, in particular, segregates direct sound-source information from the mixture of reverberant echoes that characterize the surrounding environment. Here, we used magnetoencephalography (MEG) to characterize the time courses of auditory source and space perception in the human brain. We found that the brain responses to spatial extent in reverberant environments were separable from those to the sounds that produced the reverberations and robust to variations in those sounds. Our results demonstrate the existence of dedicated neural mechanisms that separately process auditory reverberations and sources within the first few hundred milliseconds of hearing.

## Introduction

Imagine walking into a cathedral at night. Even in darkness, the passage from the narrow entryway to the large nave is immediately apparent. The reverberations produced by multiple echoes of footfalls, speech, and other sounds produce a percept of the surrounding space, distinct from the sound sources that created the echoes.

Much prior work in audition has investigated the spatial localization and perceptual organization of sound sources (Middlebrooks and Green, 1991; Bregman, 1994; Blauert, 1997; Bizley and Cohen, 2013). The surrounding environment of a sound source (i.e., the various surfaces from which reverberant reflections arise) has primarily been characterized by its effects on sound-source perception. The auditory system typically works to counteract the distorting effects of reverberation from interior surfaces, facilitating perceptual robustness of stimulus spatial position (Litovsky et al., 1999; Shinn-Cunningham, 2003; Devore et al., 2009; Brown et al., 2015), speaker identity (Brandewie and Zahorik, 2010; Watkins and Raimond, 2013), or estimated loudness (Stecker and Hafter, 2000; Zahorik and Wightman, 2001). Neurons in the auditory cortex have been shown to represent denoised or dereverberated versions of speech sounds even when presented under those distorting conditions (Mesgarani et al., 2014). Yet beyond being an acoustic nuisance to overcome, reverberations themselves provide informative cues about the environment. Humans perceive cues such as the ratio of direct to reverberant acoustic energy (DRR) to estimate sound-source distances (Mershon and King, 1975; Bronkhorst and Houtgast, 1999; Zahorik, 2001; Zahorik et al., 2005; Kolarik et al., 2016), and reverberation time (RT, a measure of reverberant energy decay) to estimate the sizes of enclosed rooms (McGrath et al., 1999; Hameed et al., 2004; Pop and Cabrera, 2005; Cabrera and Pop, 2006; Lokki et al., 2011; Kaplanis et al., 2014).

Direct and reflected environmental sounds usually arrive at the ear in a single stream; their perceptual separation is an underdetermined computational problem whose resolution by the auditory system remains unclear. Recent behavioral work suggests that the auditory system performs a scene analysis operation in which natural reverberation is separated from the originating sound source and analyzed to extract environmental information (Traer and McDermott, 2016). The neural basis of that operation, however, remains largely unexplored.

Here, to investigate the auditory coding of environmental space in the human brain, we recorded magnetoencephalography (MEG) responses to auditory stimuli comprising sounds enclosed by reverberant spaces. We operationalized spaces as the auditory room impulse response (RIR) of real-world spaces of different spatial extent (small to large rooms). The stimuli were constructed by convolving brief anechoic impact sounds of different objects with the spatial RIRs, allowing us to vary spatial extent and type of source independently. We hypothesized that both the sound source and the type of spaces could be separably decoded from the neural responses to naturalistic reverberant sounds. We found that neuromagnetic responses to spatialized sounds were readily dissociable into representations of the source and its reverberant enclosing space and that these representations were robust to environmental variations. Our MEG results constitute the first neuromagnetic marker of auditory spatial extent, dissociable from sound-source discrimination, suggesting that sound sources and auditory space are processed discretely in the human brain.

## Materials and Methods

We conducted two MEG experiments. Experiment 1 aimed to investigate whether auditory space and source representations are encoded in MEG signals and whether they are dissociable from each other. Experiment 2 was a control study examining whether neural representations reflect the timing of neural operations or low-level stimulus properties.

### Experiment 1: separating MEG signatures of sound sources and reverberant spaces

#### Participants

We recruited 14 healthy volunteers (nine females, age mean ± SD = 27.9 ± 5.2 years) with self-reported normal hearing and no history of neurologic or psychiatric disease. Participants were compensated for their time and provided informed consent in accordance with guidelines of the MIT Committee on the Use of Humans as Experimental Subjects (COUHES).

#### Stimuli

Stimuli were recordings of three different brief monaural anechoic impact sounds (hand pat, pole tap, and ball bounce), averaging 176 ms in duration. Each sound was convolved with three different monaural RIRs corresponding to real-world spaces of three different sizes, yielding a total of nine spatialized sound conditions. The RIRs were selected from a set described in detail in (Traer and McDermott, 2016). Briefly, the RIRs were measured by recording repeated Golay sequences broadcast from a portable speaker and computing the impulse response from the averaged result (Zhou et al., 1992). The use of Golay sequences allowed ambient and transient noise from busy real-world sites to be averaged out of the final response. The speaker-recorder relationship was constant at ∼1.5 m straight ahead; thus, differences between IRs did not encode variations in egocentric spatial position (azimuth, elevation, or distance) relative to the virtual sound sources in our stimuli. Rooms consisted of three real-world everyday spaces, a kitchen, a hallway, and a gym, with estimated volumes (based on room boundary dimensions) of ∼50, 130, and 600 m^3^. Reverberation times (RT_60_, the time for an acoustic signal to drop by 60 dB) of the small-, medium-, and large-space RIRs were 0.25, 0.51, and 0.68 s, respectively, averaged across frequencies from 20 Hz to 16 kHz.

#### MEG testing protocol

We presented stimuli to participants diotically through tubal-insert earphones (Etymotic Research, Elk Grove Village) at a comfortable volume, ∼70 dB SPL. Stimulus conditions were presented in random order (Psychophysics Toolbox; RRID: SCR_002881) with stimulus onset asynchronies (SOAs) jittered between 2000 and 2200 ms. Every three to five trials (four on average), a deviant vigilance target (brief speech sound) was presented, prompting participants to press a button and blink. SOAs between vigilance target and the following stimulus were 2500 ms. Target trials were excluded from analysis. Each experimental session lasted ∼65 min and was divided into 15 runs containing 10 trials from each condition, for a total of 150 trials per condition in the entire session.

#### Behavioral testing protocol

In our MEG scanning protocol, we used a passive-listening paradigm to avoid contamination of brain signals with motor-response artifacts. Thus, to test explicit perceptual judgments of the auditory scene stimuli, we conducted separate behavioral tests of space and sound-source discrimination. Participants (*N* = 14) listened to sequential pairs of the stimuli described above, separated by 1500-ms SOA. In separate blocks, participants made speeded same-different judgments on the sound sources or spaces in the stimulus pairs. Condition pairs and sequences were counterbalanced for each participant, and the order of source- and space-discrimination blocks was counterbalanced across participants. Over the course of four blocks lasting ∼40 min, participants completed a total of 36 trials per category. We collected reaction time and accuracy data from participants’ responses.

#### MEG data acquisition

MEG recordings were obtained with an Elekta Neuromag TRIUX system (Elekta), with continuous whole-brain data acquisition at 1 kHz from 306 sensors (204 planar gradiometers; 102 magnetometers), filtered between 0.3 and 330 Hz. Head motion was continuously tracked through a set of five head-position indicator coils affixed to the participant’s head.

#### MEG preprocessing and analysis

Data were motion compensated and spatiotemporally filtered offline (Taulu et al., 2004; Taulu and Simola, 2006) using Maxfilter software (Elekta). All further analysis was conducted using a combination of Brainstorm software (Tadel et al., 2011; RRID: SCR_001761) and Matlab (Natick; RRID: SCR_001622) in-house analysis scripts. We extracted epochs for each stimulus presentation with a 200-ms prestimulus baseline and 1000-ms poststimulus onset, removed the baseline mean from each sensor, and applied a 30-Hz low-pass filter.

#### MEG multivariate analysis

**Figure 1.**
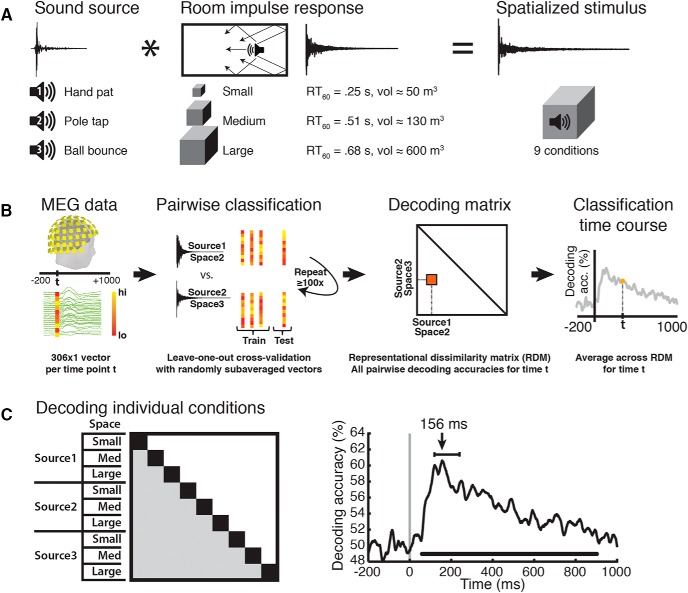
Stimulus conditions, MEG classification scheme, and single-sound decoding time course. ***A***, Stimulus design. Three brief sounds were convolved with three different RIRs to produce nine sound sources spatialized in reverberant environments. ***B***, MEG pattern vectors were used to train an SVM classifier to discriminate every pair of stimulus conditions (three sound sources in three different space sizes each). Decoding accuracies across every pair of conditions were arranged in 9 × 9 decoding matrices, one per time point *t*. ***C***, Averaging across all condition pairs (shaded matrix partition) for each time point *t* resulted in a single-sound decoding time course. Lines below time course indicates significant time points (*N* = 14, cluster-definition threshold, *p* < 0.05, 1000 permutations). Decoding peaked at 156 ms; error bars represent 95% CI.

To determine the time course of reverberant space and source discrimination, we analyzed MEG data using a linear support vector machine (SVM) classifier (Chang and Lin, 2011; RRID:SCR_010243; http://www.csie.ntu.edu.tw/~cjlin/libsvm/). For each time point *t*, the MEG sensor data were arranged in a 306-dimensional pattern vector for each of the M = 150 trials per condition (Fig. 1*B*). To increase SNR and reduce computational load, the M single-trial pattern vectors per condition were randomly subaveraged in groups of k = 10 to yield M/k subaveraged pattern vectors per condition. We then used a leave-one-out cross-validation approach to compute the SVM classifier performance in discriminating between every pair of conditions. The whole process was repeated K = 100 times, yielding an overall classifier decoding accuracy between every pair of conditions for every time point *t* (Fig. 1*B*).

The decoding accuracies were then arranged into 9 × 9 representational dissimilarity matrices (RDMs; Kriegeskorte et al., 2008), one per time point *t,* indexed by condition and with the diagonal undefined. To generate the single-sound decoding time course (Fig. 1*C*), a mean accuracy was computed from the individual pairwise accuracies of the RDM for each time point.

**Figure 2.**
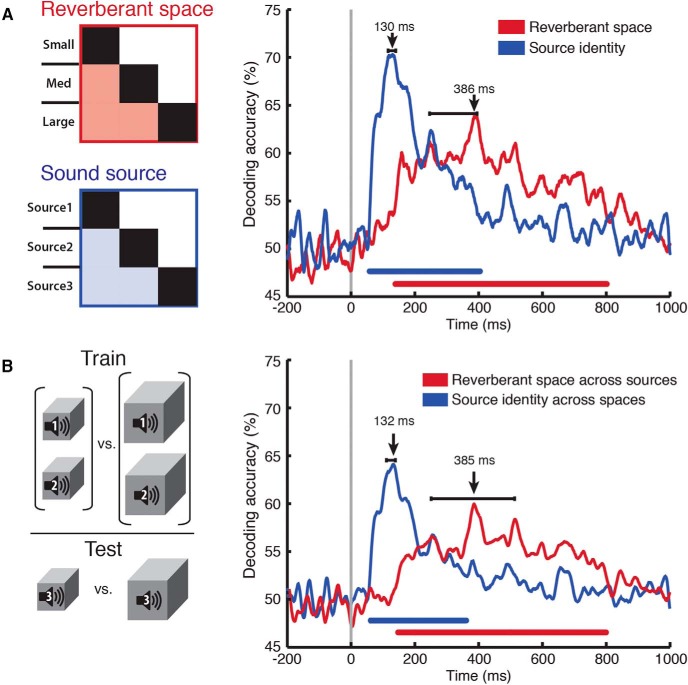
Separable space and source identity decoding. ***A***, Individual conditions were pooled across source identity (left, top) or space size (left, bottom) in separate analyses. Classification analysis was then performed on the orthogonal stimulus dimension to establish the time course with which the brain discriminated between space (red) and source identity (blue). Sound-source classification peaked at 130 ms, while space classification peaked at 386 ms. Significance indicators and latency error bars on plots same as in Figure 1. ***B***, Space was classified across sound sources and vice versa. Left panel, Cross-classification example in which a classifier was trained to discriminate between spaces on sound sources 1 and 2, then tested on space discrimination on source 3. Right panel, Sound-source cross-classification example in which a classifier was trained to discriminate between sound sources on space sizes 1 and 2, then tested on sound-source discrimination on space 3. ***B***, Results from all nine such pairwise train-test combinations were averaged to produce a classification time course in which the train and test conditions contained different experimental factors. Sound-source cross-classification peaked at 132 ms, while space cross-classification peaked at 385 ms. Significance bars below time courses and latency error bars same as in Figure 1.

For reverberant space decoding, conditions were pooled across the three sound-sources, resulting in 3M trials for each space. An SVM classifier was trained to discriminate between every pair of spaces and results were averaged across all three pairs. Decoding procedures were similar as above, but subaveraging was increased to k = 30 and repetitions to K = 300. For sound-source decoding, we pooled across the three spaces and performed the corresponding analyses (Fig. 2*A*).

Statistical significance of MEG decoding time courses was determined with permutation tests against the null hypothesis of chance-level (50%) MEG decoding accuracy. For each of 1000 permutations, each time point per participant was randomly multiplied by +1 or -1 to produce an empirical distribution of decoding accuracies from which *p* values could be derived. Cluster-size inference (Maris and Oostenveld, 2007) was used to control for multiple comparisons, with the cluster-definition threshold set at *p* = 0.05 (one-sided). Clusters were reported based on exceeding the 95% of the maximal cluster-size distribution. 95% confidence intervals (CIs) for onset and peak latencies were determined by bootstrapping the participants 1000 times and repeating the analysis to obtain empirical distributions.

#### MEG cross-classification analysis

To determine the robustness of space size and sound-source representations to environmental variation, we performed a cross-classification analysis in which different orthogonal experimental factors were assigned to training and testing sets. For example, the cross-classification of reverberant space (Fig. 2*B*) was conducted by training the SVM classifier to discriminate spaces on two sound sources and testing it on the third sound source. This analysis was repeated for all such train-test combinations and the results were averaged to produce the final cross-classification accuracy plots. SVM decoding was performed similarly to the single-condition analyses, but the training set had 2M trials, subaveraging was set to k = 20, and repetitions to K = 150. Cross-classification of sound-source identity across space sizes was performed with corresponding analyses.

#### MEG spatial (sensorwise) analysis

**Figure 3.**
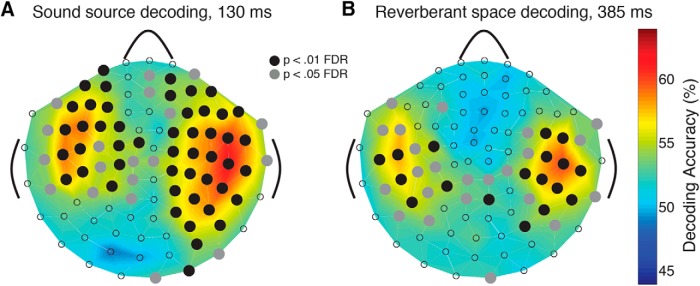
Sensorwise decoding of source identity and space size. MEG decoding time courses were computed separately for 102 sensor locations yielding decoding sensor maps. ***A***, Sensor map of sound source decoding at the peak of the effect (130 ms). ***B***, Sensor map of space size decoding at the peak of the effect (386 ms). Significant decoding is indicated with a black circle over the sensor position (*p* < 0.01; corrected for false discovery rate (FDR) across sensors and time).

The above analyses used signals from the entire suite of MEG sensors to maximize information for decoding sources and spaces. To characterize the spatial distribution of the decoding time course, we conducted a sensorwise analysis of the MEG-response patterns. Specifically, since the 306 MEG sensors are physically arranged in 102 triplets (each triplet consisting of one magnetometer and two gradiometers in the same location), we repeated the multivariate analyses described above at each of the 102 sensor locations but using a three-dimensional (rather than 306-dimensional) pattern vector for each location. This yielded a 9 × 9 RDM of pairwise classification accuracies at each sensor location and at each time point. Thus, rather than the single whole-brain decoding time course shown in Figures 1 and 2, we generated 102 decoding time courses, one for each sensor triplet location, visualized as sensor maps (Fig. 3). Statistically significant decoding accuracies were determined via permutation analysis (*N* = 14, sign permutation test with 1000 samples, *p* < 0.01, corrected for FDR across sensor positions at each time point).

#### MEG temporal generalization analysis

**Figure 4.**
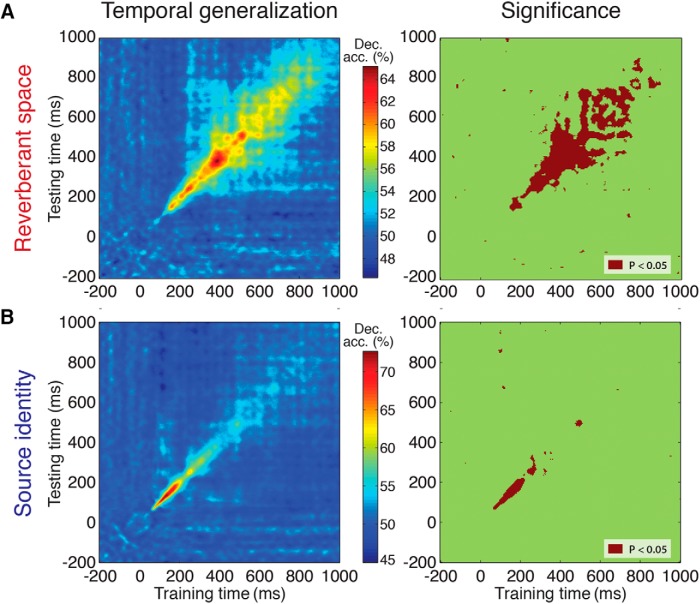
Temporal generalization matrix of auditory source and space decoding time courses. Left column shows the generalized decoding profiles of space (***A***) and source (***B***) decoding. Right column shows the statistically significant results (*t* test against 50%, *p* < 0.05, FDR corrected).

The above analyses trained and tested each SVM classifier at a unique time point, which necessarily limits information about the persistence or transience of neural representations. Thus, to further interrogate the temporal dynamics of reverberant space and source identity processing, we generalized the above analysis by training each classifier at a given time point *t* and testing against all other time points *t*’. This yielded a two-dimensional temporal generalization (“time-time”) matrix, where the *x*- and *y*-axes index the training and testing time points, respectively, of the classifiers (Fig. 4; King and Dehaene, 2014). Statistical significance maps for each matrix were generated via *t* test across participants for each time-time coordinate, *p* < 0.05, FDR corrected.

#### Analysis of stimulus properties

We generated time-frequency cochleograms from each stimulus using a Matlab-based toolbox (Slaney, 1998; Ellis, 2009) that emulates the filtering properties of the human cochlea. Each wave form was standardized to 44,100 samples and passed through a gammatone filterbank (64 subbands, center frequencies 20-20,000 Hz), summing the energy within overlapping 20-ms windows in 5-ms steps. The cochleograms were then correlated pairwise at each time point, with 64-element pattern vectors comprising the energy per frequency subband in each 5-ms bin. The resulting Pearson correlation coefficients were then subtracted from 1 and averaged to compute the stimulus dissimilarity measure for that time point (Fig. 6*A*). Repeating this analysis across time points yielded the overall cochleogram-based dissimilarity curve (Fig. 6*B*). The same analysis was performed on conditions pooled by source identity and space size (Fig. 6*C*) to produce separate source and space dissimilarity time courses. Significance of the peak mismatches with the MEG decoding peaks was determined via examination of confidence intervals from bootstrapping MEG peak latencies 10,000 times.

### Experiment 2: controlling for stimulus duration

For experiment 2, we recorded MEG data while participants (*N* = 16) listened to stimuli comprising the same impact sounds as in the main experiment, but repeated 10 times at 200-ms intervals, and then convolved with the same RIRs used in the main experiment. The 2-s wave form was then linearly ramped up for 1 s and down for 1 s to avoid strong attacks at the onset of the stimulus (Fig. 7*A*). Consequently, each stimulus contained its source and spatial information distributed throughout the 2000-ms repetition window. We reasoned that if peak decoding latencies reflected the neural process underlying space perception, they would not be strongly yoked to stimulus temporal structure and would thus not be strongly shifted compared with experiment 1. By contrast, MEG decoding signatures yoked to the stimulus temporal structure should not only last throughout the duration of the stimulus, but peak at ∼1000 ms, the peak of the stimulus amplitude envelope.

Because of the longer stimulus duration, experimental sessions were ∼10 min longer than those in the main experiment. The MEG time series extracted from the neural data spanned 2701 rather than 1201 time points, from -200 to +2500 ms relative to stimulus onset. All other parameters (organization of stimulus conditions, task, presentation procedure, significance calculations) remained the same as in experiment 1. In computing bootstrapped peak latencies, we included only the time during which the stimulus was actively playing, between 0 and 2000 ms.

All statistical analyses are summarized in Table 1, with superscript letters in specific Results indicating rows in the table.

**Table 1.** Summary of key statistical tests

Line	Data structure	Type of test	95% confidence intervals
a	None assumed: classification accuracy over time	Bootstrap *N* = 14 participants 1000 times to obtain empirical distribution of significant decoding onset	Onset CI: 12–64 ms
b	None assumed: classification accuracy over time	Bootstrap *N* = 14 participants 1000 times to obtain empirical distribution of significant decoding peak	Peak CI: 119–240 ms
c	None assumed: classification accuracy over time	Bootstrap *N* = 14 participants 1000 times to obtain empirical distribution of significant sound-source decoding onset	Onset CI: 37–60 ms
d	None assumed: classification accuracy over time	Bootstrap *N* = 14 participants 1000 times to obtain empirical distribution of significant sound-source decoding peak	Peak CI: 116–140 ms
e	None assumed: classification accuracy over time	Bootstrap *N* = 14 participants 1000 times to obtain empirical distribution of significant space decoding onset	Onset CI: 71–150 ms
f	None assumed: classification accuracy over time	Bootstrap *N* = 14 participants 1000 times to obtain empirical distribution of significant space decoding peak	Peak CI: 246–395 ms
g	None assumed: onsets of source and space decoding	Compare bootstrapped empirical distribution of space decoding onset with mean source decoding onset	Space onset CI: 71–150 ms
h	None assumed: peaks of source and space decoding	Compare bootstrapped empirical distribution of space decoding peak with mean source decoding peak	Space peak CI: 246–395 ms
i	None assumed: cross-classification accuracy over time	Bootstrap *N* = 14 participants 1000 times to obtain empirical distribution of significant sound-source cross-decoding peaks	Onset CI: 40–63 ms Peak CI: 111–139 ms
j	None assumed: cross-classification accuracy over time	Bootstrap *N* = 14 participants 1000 times to obtain empirical distribution of significant space cross-decoding peaks	Onset CI: 125–356 ms Peak CI: 251–513 ms
k	None assumed: MEG-behavior correlations	Bootstrapping *N* = 14 pool, 10,000 iterations of Spearman correlation between behavioral reaction time and MEG peak latency	CI: .227–.895
l	None assumed: MEG-behavior correlations	Bootstrapping *N* = 14 pool, 10,000 iterations of Spearman correlation between behavioral accuracy and MEG peak accuracy	CI: .325–.795
m	None assumed: empirical distribution of source decoding peak	Compare bootstrapped empirical distribution of source decoding peak with source dissimilarity peak	Peak CI: 116–140 ms
n	None assumed: empirical distribution of space decoding peak	Compare bootstrapped empirical distribution of space decoding peak with mean space dissimilarity peak	Peak CI: 246–395 ms
o	Normal distribution: MEG-model correlations over time points	Paired *t* test between mean correlations	Mean difference CI: 0.0470–0.0507
p	None assumed: classification accuracy over time	Bootstrap *N* = 16 participants 1000 times to obtain empirical distribution of significant source decoding onset	Source peak CI: 96–312 ms
q	None assumed: classification accuracy over time	Bootstrap *N* = 16 participants 1000 times to obtain empirical distribution of significant source decoding onset	Space peak CI: 71–790 ms

## Results

### Experiment 1

#### Auditory representations discriminated sound sources and reverberant spaces with temporally dissociable and generalizable decoding trajectories

We applied SVM to decode every pair of conditions (Fig. 1*B*; Carlson et al., 2013; Cichy et al., 2014, 2015). The pairwise decoding accuracies were averaged to create an overall single-condition classification time course. Classification performance increased sharply from chance levels shortly after stimulus onset, reaching significance at 59 ms (95% CI: 12–64 ms)^a^ and peaking at 156 ms (119–240 ms)^b^. These results indicate the MEG signal was able to reliably distinguish between individual stimulus conditions.

To dissociate the neuronal dynamics of space size and sound-source discrimination, we repeated this analysis, but pooled trials across the corresponding conditions before decoding. This resulted in 3 × 3 RDM matrices (Fig. 2*A*, left), and averaging across the shaded regions produced the time courses of space size (red) and source identity (blue) decoding (Fig. 2*A*, right). The transient nature of source discrimination, reaching significance at 57 ms (37–60 ms)^c^ and peaking at 130 ms (116–140 ms)^d^, is in sharp contrast to the slower, sustained response of the reverberant space decoding time course, which exhibited a significantly later decoding accuracy significance onset [138 ms (71–150) ms)^e^] and peak [386 ms (246–395 ms)^f^; onset latency difference, *p* = 0.01^g^; peak latency difference, *p* < 0.001^h^]. This suggests that sound-source information is discriminated early by the auditory system, followed by reliable reverberant space discrimination. In experiment 2, we found that sources and spaces were still decodable when all stimuli were controlled for duration, suggesting that the timing is not solely dependent on stimulus duration (Fig. 8).

Stable source and reverberant space representations should be tolerant to other changing properties in a scene, such as low-level spectral differences between sound sources or spectral modulation differences between RIRs. Thus, we conducted a cross-classification analysis in which we assigned space size conditions from two sources to a training set, and the size conditions from the remaining source to a testing set (Fig. 2*B*, left). Results from all such train/test combinations were averaged to produce a measure of space size information generalized across sound sources, with sound sources not overlapping between training and testing sets. We also performed an analogous analysis to cross-classify sound sources across spaces. The results (Fig. 2*B*, right) indicate time courses consistent with those in the pooled analysis, with source cross-decoding onset at 57 ms (40–63 ms), peaking at 132 ms (111–139 ms)^i^; and space cross-decoding onset at 148 ms (125–136 ms), peaking at 385 ms (251–513 ms)^j^. This demonstrates that the neural representations of reverberant space and sound source are robust to variations in an orthogonal dimension.

#### Source identity and reverberant space were best decoded by bilateral temporal sensors

To determine the spatial distribution of the decoding response, we repeated the main analysis on sensor clusters in 102 distinct locations across the MEG helmet. This analysis revealed that the bulk of significant decoding performance (*p* < 0.01, FDR corrected across sensors at each time point) was concentrated in sensors over bilateral temporal regions (Fig. 3). While the spatial interpretation of such an analysis is limited in resolution, the results implicate bilateral auditory cortical regions as generators of the underlying neural signals distinguishing sound source and space size information.

#### Dynamics of reverberant space representations are slower and more sustained compared with sound-source representations

To examine the temporal dynamics of source and space representations, we conducted a temporal generalization analysis (Cichy et al., 2014; King and Dehaene, 2014) in which a classifier trained at one time point was tested on all other time points. This produced a two-dimensional matrix showing generalized decoding profiles for space and source identity (Fig. 4). The results suggest differences in processing dynamics: the narrow “diagonal chain” pattern shown for source identity decoding in Figure 4*B* indicates that classifiers trained at a time point *t* only generalize well to neighboring time points; by contrast, the space profile (Fig. 4*A*) exhibits a broader off-diagonal decoding regime, indicating that classifiers were able to discriminate between space conditions over extended time periods. This suggests that reverberant space representations are mediated by more metastable, sustained underlying neural activity, compared with transient, dynamic activity mediating sound-source representations (King and Dehaene, 2014).

#### MEG decoding dynamics predict relative timing and accuracy of behavioral judgments

**Figure 5.**
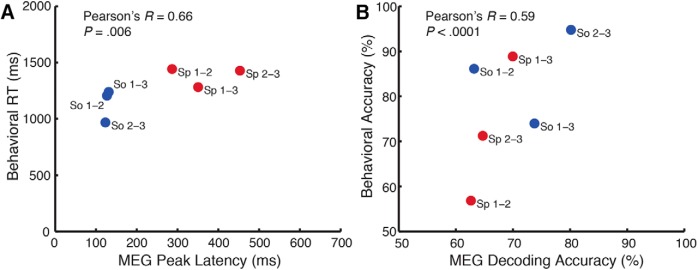
Behavior correlates with MEG decoding data. Assessment of linear relationships between response times and MEG peak decoding latencies (***A***), as well as behavioral and decoding accuracies (***B***). Bootstrapping the participant sample (*N* = 14, *p* < 0.05) 10,000 times revealed significant correlations between RT and latency (*r* = 0.66, *p* = 0.0060) and behavioral and decoding accuracy (*r* = 0.59, *p* < 0.0001). Individual condition pairs are denoted by source (So; red) or space (Sp; blue) labels, with numerals indicating which conditions were compared. For space conditions: 1, small; 2, medium; 3, large. For source conditions: 1, hand pat; 2, pole tap; 3, ball bounce.

To extract behavioral parameters that could be compared with the dynamics of the MEG signal, we binned all trials into appropriate source or space comparison categories (e.g., Space1 vs Space2, Source1 vs Source3, etc.). Within each category, we computed each participant’s mean accuracy and mean response time (mean RT estimated by fitting a γ distribution to the response time data; Palmer et al., 2011). This yielded mean accuracies and RTs in three source-comparison and three space-comparison conditions, analogous to the pooled MEG decoding analysis. Behavioral accuracies and RTs were then correlated with MEG peak decoding accuracies and peak latencies, respectively. Significance and confidence intervals were determined by bootstrapping the behavioral and MEG participant pools 10,000 times. Behavioral RTs and peak latencies were significantly correlated (*r* = 0.66, *p* = 0.0060)^k^, as were behavioral accuracies and peak decoding accuracies (*r* = 0.59, *p* < 0.0001)^l^ (Fig. 5).

#### MEG decoding peaks are not explained by stimulus temporal structure

**Figure 6.**
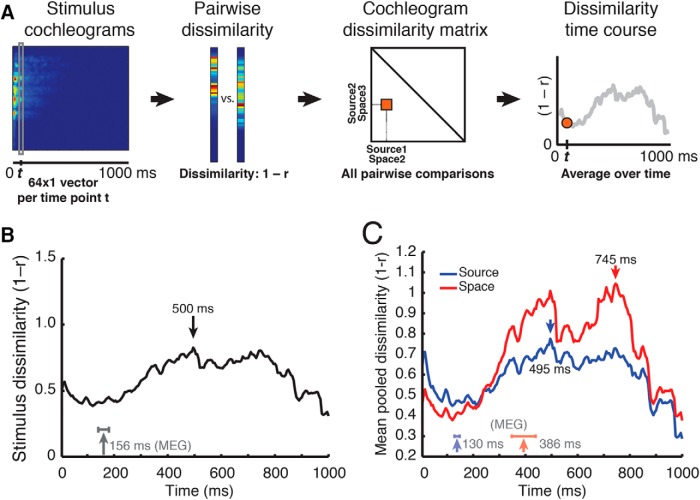
Stimulus dissimilarity analysis based on cochleogram data. ***A***, Cochleograms were generated for each stimulus, discretized into 200 5-ms bins and 64 frequency subbands. Each cochleogram thus comprised 200 64 × 1 pattern vectors. For each pair of stimuli, pattern vectors across frequency subbands were correlated at corresponding time points and subtracted from 1. ***B***, Overall cochleogram-based dissimilarity. The final dissimilarity value at time *t* is an average of all pairwise correlations at that time point. Peak overall cochleogram dissimilarity occurred at 500 ms; peak MEG dissimilarity (decoding accuracy) is shown for comparison. ***C***, Pooled cochleogram-based dissimilarity across space size and source identity. Pairwise correlations were performed and averaged analogously to pooled decoding analysis. MEG pooled decoding peaks for source identity and space size are shown for reference; corresponding stimulus dissimilarity peaks were significantly offset (*p* < 0.05 for both source identity and space).

To determine the extent to which the MEG signal could be explained by low-level responses to stimulus properties, we generated and correlated cochleograms pairwise from each stimulus condition. This analysis yielded an overall cochleogram-based dissimilarity curve (Fig. 6*B*) and, when performed on conditions pooled by source identity and space size, separate source and space dissimilarity curves (Fig. 6*C*). The cochleogram-based dissimilarity peaks (source, 495 ms; space, 795 ms) were significantly mismatched with the MEG decoding peaks (*p* < 0.001 for both source^m^ and space^n^ via comparing to bootstrapped MEG peak latencies), a disparity that suggests the neural signal reflects processing other than the temporal structure of the stimulus.

**Figure 7.**
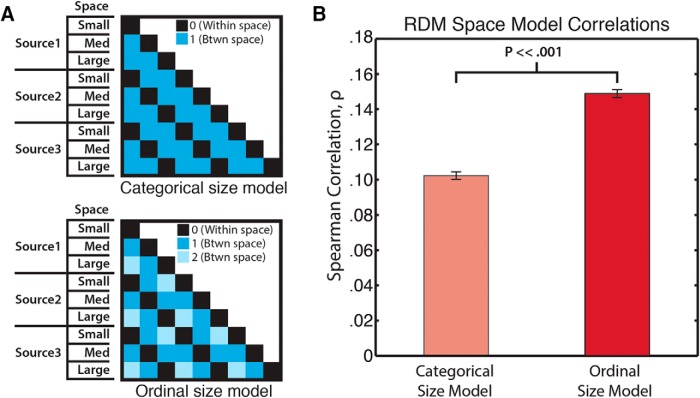
Comparison of MEG neural representations to a categorical versus an ordinal scene size model. Representational dissimilarity matrices (RDMs) of a categorical and an ordinal model (***A***) were correlated with the MEG data from 138–801 ms (the temporal window of significant space size decoding) to assess the nature of MEG scene size representations. ***B***, Results indicate the MEG representations have significantly higher correlation with the ordinal than the categorical scene size model. Spearman correlation coefficients ρ were averaged across time points in the temporal window. Error bars represent ±SEM.

#### Reverberant spaces are encoded in a stepwise size progression

The MEG space decoding results shown in Figure 2*B* could be suggestive of an encoded size scale (i.e., a small-to-large progression), or they could simply reflect a generic category difference between the three space conditions. To evaluate whether MEG responses were consistent with ordinal versus categorical spatial extent coding, we devised simple models of space representation in the form of RDMs that reflected the hypothesized space extent representations. That is, each pairwise representational distance in the model 9 × 9 condition matrix was either 0 or 1, reflecting a within versus between separation (categorical space model), or 0, 1, or 2 reflecting a pure ordinal separation between space conditions respective of sound-source identity (ordinal space model). We then correlated (using Spearman rank to capture ordinal relationships) the model RDMs with the brain-response RDMs at every time point between the first and last time point of significant space decoding in the pooled analysis (138–801 ms poststimulus onset). Figure 4 shows that an ordinal space model correlates significantly more strongly with the neural data than a categorical model (663 time points, paired *t* test, *p* < 0.00001)^o^, suggesting an ordinal representation of spatial size.

### Experiment 2

#### Temporally extended stimuli elicit decoding dynamics similar to those of single-pulse stimuli

**Figure 8.**
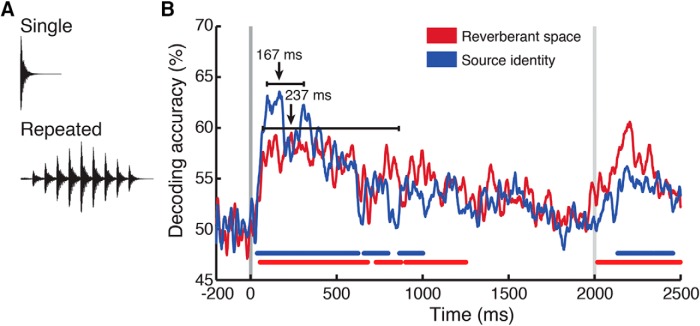
Space and sound source decoding with repetition-window stimuli. ***A***, Representative waveforms of single and repeated stimuli. Repeated stimuli were produced by concatenation of anechoic stimuli, followed by RIR convolution and linear amplitude ramping. ***B***, Source (blue) and space (red) decoding. Sound-source classification peaked at 167 (96-312) ms, while space classification peaked at 237 (71-790) ms. Color-coded lines below time courses indicate significant time points, as in experiment 1; latency error bars indicate bootstrapped confidence intervals as in experiment 1. Gray vertical lines indicate stimulus onset and approximate offset.

To examine whether peak decoding latencies reflect the timing of a neural operation or depend strictly on stimulus properties, we conducted a second MEG experiment examining the effect of longer (2000 ms) stimulus durations on decoding latencies. As shown in Figure 8*B*, sound-source decoding peaked at 167 ms (96–312 ms)^p^, while space decoding peaked at 237 ms (71–790 ms)^q^. Responses remained significant throughout much of the stimulus duration but peaked early on, despite the amplitude envelope peaking in the middle of the stimulus, 1000 ms postonset. Thus, the neuromagnetic decoding signal reflects processing dissociable from the source and space information distributed throughout the longer stimulus.

## Discussion

We investigated the neural representation of transient sound sources and their reverberant environments using multivariate pattern analysis (Carlson et al., 2013; Cichy et al., 2014) on MEG brain responses to spatialized sounds. Our results showed that overall individual sound conditions were decoded starting at ∼60 ms poststimulus onset, peaking at 156 ms. Next, we characterized the separate neural time courses of source- and RIR-specific discrimination. These decoding profiles emerged with markedly different time courses, with the source discrimination time course exhibiting a rapid-onset transient response peaking at 130 ms, and the space discrimination time course ramping up more gradually to peak at 386 ms. Generalization of the responses across low-level variations was revealed by a cross-classification analysis in which training and testing trials contained different experimental factors. This suggests that these representations are tolerant to differences in amplitude envelope and spectral distributions, which accompany environmental changes commonly encountered in real-world situations. A sensorwise decoding analysis showed that bilateral temporal cortical areas contributed most heavily to the decoding performance. MEG decoding peaks were significantly correlated with behavioral responses collected separately. Finally, the MEG decoding signal did not share the temporal profile of interstimulus correlations and peaked at similar times even when the stimulus was temporally extended, suggesting that it is separate from the stimulus temporal structure. Taken together, the results suggest that the MEG decoding time series capture the neural processes mediating extraction of sound source and reverberant information from naturalistic stimuli.

### Dissociable and independent decoding time courses

The pooled and cross-classified source and space decoding time courses (Figs. 2, 3) indicate that reverberant space and sound-source representations are dissociable and independent in the brain. The early sound-source decoding peak suggests that source or object identity information is primarily carried by the direct sound and accompanying early reflections (the first, most nearby surface echoes; Hameed et al., 2004). By contrast, the later space decoding peak and concurrent falloff in source decoding suggest that reverberant decay primarily carries spatial extent information. This is broadly consistent with physical properties of large spaces, i.e., the longer propagation time for reflections to reach the listener, as well as longer RT_60_ reverberant decay times, in large spaces. However, even when source and space information was distributed throughout the stimuli equated for duration in experiment 2, decoding peaks maintained a similar window of absolute and relative timing (Fig. 8), suggesting a consistent neural basis for the decoding time courses. However, testing other temporally extended nonimpulsive stimuli (e.g., speech, other environmental or synthetic sounds) constitutes a fruitful avenue for future work.

### Bilateral temporal decoding loci

We observed a bilateral temporal decoding response to the stimulus conditions for space sizes as well as sound-source identities. While the spatial resolution of the sensorwise analysis cannot determine the exact loci of the signal sources, our results are consistent with a bilateral (Alain et al., 2001; Arnott et al., 2004) account of a spatial auditory processing stream, distinguishable from auditory object identification as early as nonprimary auditory cortical regions (Ahveninen et al., 2006, 2013). Room size judgments have been found to be correlated with sound-source distance judgments (Kolarik et al., 2013); while this may suggest a shared mechanism, it is unlikely to indicate distance as a direct proxy for size: the impulse responses in our stimuli kept sound-source distance constant, and the right-lateralized temporal processing of egocentric distance (Mathiak et al., 2003) is inconsistent with the bilateral decoding pattern in our results. Still, a source/reverberant space separation operation by the auditory system would facilitate computing DRR for distance perception. Our data do not address this question directly but, along with Traer and McDermott (2016), suggest an intriguing counterpoint to interpretations that DRR computation is ill-posed and thus unlikely (Kopco and Shinn-Cunningham, 2011), or is bypassed via perception of other covarying cues (Larsen et al., 2008).

It has been speculated (Epstein, 2011) that spatial auditory scenes may be processed by the retrosplenial complex (RSC) and parahippocampal place area (PPA), occipital and ventral temporal brain regions known to be responsive to visually and haptically presented scene attributes (Wolbers et al., 2011; Park et al., 2015). The present sensorwise analysis most clearly implicates auditory-specific cortices in coding space size, although it does not preclude RSC and PPA. In addition to further examining this question, future work could compare the neural responses of blind and sighted listeners: people who are blind or visually impaired tend to weight echoes more heavily in perceiving their environments, whether passively listening (Després et al., 2005; Dufour et al., 2005) or actively echolocating (Teng et al., 2012; Kolarik et al., 2014; Thaler, 2015). Thus, given increased reliance on auditory information in blindness, frameworks of neuroplasticity espousing fundamental modality independence in neural function (Peelen and Kastner, 2009) suggest that RSC similarly represents auditory spatial scenes in blind persons.

### Dynamics in relation to previous electrophysiological work

Most prior neuroimaging work on spatial auditory perception, including studies that used reverberant stimuli, measured brain responses to sound-source properties, rather than properties of the enclosing space. The space- and source-decoding peak latencies found here are similar to that of numerous evoked neuromagnetic responses such as the mismatch negativity (Mathiak et al., 2003; King et al., 2014) and N1m response (Palomäki et al., 2005). This generally suggests that the neural activity underlying these evoked components may also be driving the source-specific MEG decoding performance, while low-level, preattentive-evoked responses with shorter latencies, such as the P50 (Mathiak et al., 2003), do not reflect neural activity that distinguishes among the spatial conditions, despite overlapping origins in bilateral temporal cortex (Liégeois-Chauvel et al., 1994). Later responses to naturalistic spatial stimuli include elevation-related processing starting at 200 ms (Fujiki et al., 2002) and a component indexing 3D “spatiality” of sound sources (Tiitinen et al., 2006), a reverberation-mediated parameter that may share similar underlying mechanisms with the space decoding signal in our results.

### The metrics of space representation

While behavioral room size judgments have been previously shown to be driven by reverberant information, the precise relationship between volumetric spatial extent, reverberation time, and perceived room size is nonlinear and not fully understood (Mershon and King, 1975; Hameed et al., 2004; Kaplanis et al., 2014). We used a well-discriminable sequence of impulse responses from differently sized real-world spaces to establish an ordinal representation of spatial extent, but future work can more precisely characterize the metric by which the brain encodes auditory spatial extent and the interactions of monaural and binaural components of the signal. Future work may also more precisely map different acoustic components of the space signal to different neural correlates of perception, thus far explored almost exclusively in the behavioral realm (Kaplanis et al., 2014; but see Lawless and Vigeant, 2015). While spatial hearing is often considered inherently binaural, spatial properties carried by reverberation are not always so: diotic monaural stimuli have been used to judge spaces (Berkley and Allen, 1993; Traer and McDermott, 2016) and may in fact provide more salient spatial information than when listening binaurally (Shinn-Cunningham and Ram, 2003). Even when using binaural impulse responses, monaural cues in the sound are among the strongest predictors of perceived room size judgments (Pop and Cabrera, 2005; Zahorik, 2009). Still, binaural aspects of reverberation contain cues to spatially relevant percepts, such as spaciousness and apparent source width (Kaplanis et al., 2014), that our data do not address, and binaural listening may “squelch” or suppress perceived reverberation (Gelfand and Hochberg, 1976; but see Ellis et al., 2015). Finally, our RIRs were chosen from a set with RT_60_ averaging <1 s, and we did not explore effects of much longer times, which tend to correspond to larger spaces. Thus, our reverberant diotic stimuli likely reflect a subset of possible salient spatial extent cues that a real-world listener would encounter.

We note that space size as operationalized here is best defined for indoor environments; outdoor environments also have reverberant impulse responses, but their utility for judging spatial scale per se (vs sound-source distance; cf. Zahorik, 2002) is unclear. Although spatial layout can be recovered computationally from a few initial echoes (Dokmanic et al., 2013), human observers do not seem to have access to this information in real-world spaces (Calleri et al., 2016). Even in the visual scene and animal literatures, outdoor spaces have not been operationalized consistently (Kravitz et al., 2011; Park et al., 2011; Geva-sagiv et al., 2015; Vanderelst et al., 2016), and thus the scene (and scene size) construct in general remains a topic for further research. However, recent work in scene processing has established that visual environments are represented along separable and complementary dimensions of spatial boundary and content (Kravitz et al., 2011; Park et al., 2011, 2015; Vaziri et al., 2014; Cichy et al., 2016). Thus, a visual scene may be characterized by, e.g., its encompassing shape and size, as well as by the number, type, and configuration of objects it contains (Oliva and Torralba, 2001; Oliva, 2013). Given that both visual and haptic scene exploration elicits responses in scene-selective brain regions (Wolbers et al., 2011), it is reasonable to surmise (cf. Epstein, 2011) some multimodality of scene representation. As a reliable index of perceived auditory scene extent (i.e., room size), natural reverberation could thus trigger scene-specific processing in a temporal regime that overlaps with those reported in recent M/EEG studies of visual scenes (Cichy et al., 2016; Harel et al., 2016).

In sum, the current study presents the first neuromagnetic evidence for the separation of auditory scenes into source and reverberant space representations in the brain. The neurodynamic profile of the processing stream is dissociable from that of sound sources in the scene, robust to variations in those sound sources, and predicts both timing and accuracy of corresponding behavioral judgments. Our results establish an auditory basis for neuroscientific investigations of scene processing, suggest the spatial importance of the reverberant decay in perceived scene properties, and lay the groundwork for future auditory and multisensory studies of perceptual and neural correlates of environmental geometry.

## Synthesis

Reviewing Editor: Bradley Postle, University of Wisconsin Decisions are customarily a result of the Reviewing Editor and the peer reviewers coming together and discussing their recommendations until a consensus is reached. When revisions are invited, a fact-based synthesis statement explaining their decision and outlining what is needed to prepare a revision will be listed below. The following reviewer(s) agreed to reveal their identity: Andrew Kolarik

Both reviewers are enthusiastic about this work, and their suggestions can be addressed with some modifications of the text.

R1

This manuscript described the results of an MEG study that reveals how the brain represents reverberant spaces separately from sound sources. The work is excellent. It is highly innovative and will have significant impact on the field. They clearly advance understanding of the mechanisms that support perception of sound sources in realistic reverberant listening conditions. There are some significant limitations of this work, however. These include: 

1) The fact that the stimulus presentation techniques are monaural. This seems to severely limit the claims about space, given that the spatial attributes of monaural signals are dramatically different than binaural signals. From the architectural acoustic literature, it is known that various binaural aspects of reverberation are highly salient perceptually (e.g. reverberation “spaciousness” and apparent source width). It is also known that reverberation when presented monaurally, can have quite different perceptual characteristics than when presented binaurally (e.g. “binaural squelch of reverberation”). It is also clear that the natural mode of listening in a reverberant sound field for humans is a binaural mode, not monaural. The authors do recognize some of these issues on p. 20, but I think this limitation needs to be made much more clearly and earlier in the manuscript. Ideally the abstract should mention something about monaural presentation. 

2) Since only three rooms were tested, with a maximum reverberation time of 0.68s, I think some qualification of the generality of the results is warranted. There are many realistic listening spaces with much longer reverberation times. 

3) Similarly, since only three source signals were tested, and they were all impulsive in nature, I believe there should be some additional qualification of the generality of the results in this regard. The statement in the abstract “the reverberant space response was robust to variations in sound source, and vice versa, indicating a generalized response not tied to any specific source-space combination” is potentially misleading, since it is not clear that enough source-space combinations were tested to make this claim. Related: What was the rationale for testing only impulsive sounds? 

These limitations can all be easily addressed through simple manuscript revisions. 

R2

The author(s) investigated how information regarding sound sources and reverberant spaces are segregated in the human brain. Using MEG, they show that a neuromagnetic response for auditory space was found to be dissociable in terms of form and time from the response to the sound source from which the reverberant sound originated. This is a novel finding, supporting the proposition that representations of the sound source and reverberant space are dissociable within the brain. 

The findings are interesting and important, and the manuscript is on the whole well written and clear in terms of flow and language. The methods are sufficiently detailed. I believe that the findings will be of interest to a broad readership. I have a few comments that the authors may wish to address. 

General points 

The authors' findings suggest that direct sound and reverberant sound are separately represented in the brain. Although spatial extent rather than distance was the focus of the manuscript, their findings may have implications for how direct-to-reverberant energy ratio (DRR or D/R) is computed for use as a cue to sound source distance - if the brain is able to separate the direct and reverberant sound, then DRR could be calculated, and perhaps some material could be added to the paper discussing this. In any case, the use of direct vs. reverberant sound as a distance cue is touched on in the Introduction but should be explicitly named and briefly expanded upon. 

In their 2011 JASA paper, Kopčo & Shinn-Cunningham mention that “... it is very unlikely that the D/R is directly computed by the auditory system. In order to compute D/R directly, listeners would have to separate the direct sound from the reﬂected sound in the total signal... (p 1539).” Co-varying acoustic characteristics of the signal including spectrum or timing information have been put forward as an alternative to compute DRR (Larsen et al, 2008). However, do the authors' findings not suggest that the direct and reverberant sound are indeed being computed separately in the brain, at least for the transient sound sources they tested? It would be nice if the authors could add some brief comments regarding whether their findings bear on the issue of how DRR might be computed by the auditory system in the Discussion section. 

References: 

Kopčo, N., & Shinn-Cunningham, B. G. (2011). Effect of stimulus spectrum on distance perception for nearby sources. The Journal of the Acoustical Society of America, 130, 1530-1541. 

Larsen, E., Iyer, N., Lansing, C. R., & Feng, A. S. (2008). On the minimum audible difference in direct-to-reverberant energy ratio. The Journal of the Acoustical Society of America, 124, 450-461 

Specific comments 

1) Page 3 line 52: “...humans are able to perceive those cues to estimate features such as sound source distances (Mershon and King, 1975; Bronkhorst and Houtgast, 1999; Zahorik, 2001; Zahorik et al., 2005)...” As mentioned above, DRR as a cue to auditory distance should be explicitly described at this point in the manuscript. Also, suggest adding a more recent review of distance perception to the papers cited here: 

Kolarik, A. J., Moore, B. C. J., Zahorik, P., Cirstea, S., & Pardhan, S. (2016). Auditory distance perception in humans: a review of cues, development, neuronal bases and effects of sensory loss. Atten. Percept. Psycho., 78, 373-395. 

2) Page 5 line 95: “Stimuli were recordings of three different brief monaural anechoic impact sounds (hand pat, pole tap, and ball bounce)...” Please state why these stimuli were chosen. In terms of what acoustic characteristics are they different? 

3) Page 22 line 480: “While spatial hearing is often considered inherently binaural, consider the cathedral from our Introduction: the reverberant quality of its spacious interior is perceptible from the outside, even through a doorway that effectively functions as a single sound source.” I have difficulty following this, and I am not sure it is necessary. How does the doorway functioning as a single sound source relate to binaural vs. monaural spatial listening? Please rewrite for clarity, or consider removing the text. 

4) Page 20 line 440: “...temporal brain regions known to be responsive to for visually and haptically presented scene attributes...” Suggest removing “for.” 

5) Unless I have missed it, I cannot see where Table 1 is referenced in the main text.
